# FMECA Application to Intraoperative Electron Beam Radiotherapy Procedure As a Quality Method to Prevent and Reduce Patient’s Risk in Conservative Surgery for Breast Cancer

**DOI:** 10.3389/fmed.2017.00138

**Published:** 2017-08-28

**Authors:** Cristiana Vidali, Mara Severgnini, Monica Urbani, Licia Toscano, Alfredo Perulli, Marina Bortul

**Affiliations:** ^1^Department of Radiation Oncology, Azienda Sanitaria Universitaria Integrata di Trieste (ASUITS), Trieste, Italy; ^2^Department of Medical Physics, Azienda Sanitaria Universitaria Integrata di Trieste (ASUITS), Trieste, Italy; ^3^Department of Surgery, University of Trieste, Trieste, Italy; ^4^Department of Physics, University of Trieste, Trieste, Italy; ^5^Department of Medical Direction, Azienda Sanitaria Universitaria Integrata di Trieste (ASUITS), Trieste, Italy

**Keywords:** risk assessment, intraoperative electron beam radiotherapy, quality assurance, FMECA, patient safety

## Abstract

**Introduction:**

Failure Mode Effects and Criticalities Analysis (FMECA) represents a prospective method for risk assessment in complex medical practices. Our objective was to describe the application of FMECA approach to intraoperative electron beam radiotherapy (IOERT), delivered using a mobile linear accelerator, for the treatment of early breast cancer as an anticipated boost.

**Materials and methods:**

A multidisciplinary Working Group, including several different professional profiles, was created before the beginning of clinical practice in 2012, with the purpose of writing the Flow Chart and applying the FMECA methodology to IOERT procedure. Several criticalities were identified *a priori* in the different steps of the procedure and a list of all potential failure modes (FMs) was drafted and ranked using the risk priority number (RPN) scoring system, based on the product of three parameters: severity, occurrence, and detectability (score between 1 and 5). The actions aimed at reducing the risk were then defined by the Working Group and the risk analysis was repeated in 2014 and in 2016, in order to assess the improvement achieved.

**Results:**

Fifty-one FMs were identified, which represented the issues prospectively investigated according to the FMECA methodology. Considering a set threshold of 30, the evaluated RPNs show that 33 out of 51 FMs are critical; 6 are included in the moderate risk class (RPN: 31–40); 16 in the intermediate risk class (RPN: 41–50), and 11 in the high risk class (RPN: >50).

**Discussion:**

The most critical steps concerned the surgical procedure and IOERT set-up. The introduction of the corrective actions into the clinical practice achieved the reduction of the RPNs in the re-analysis of the FMECA worksheet after 2 and 4 years, respectively.

**Conclusion:**

FMECA proved to be a useful tool for prospective evaluation of potential failures in IOERT and contributed to optimize patient safety and to improve risk management culture among all the professionals of the Working Group.

## Introduction

Adjuvant radiotherapy (RT) after breast conservative surgery is currently considered the standard treatment for early breast cancer and plays an important role to reduce local recurrences (LR) and to improve disease-free and overall survival (1).

Taking into consideration that around 85% of ipsilateral breast cancer recurrences occur in the tumor bed, a local dose escalation is commonly used in addition to whole breast irradiation (2). Three randomized trials have demonstrated that the addition of a boost to the tumor bed reduces further the incidence of LR (3, 4).

Intraoperative electron beam radiotherapy (IOERT) in the treatment of early-stage breast cancer was introduced into the clinical practice at the end of the 1990s, when dedicated mobile linear accelerators (linacs) became available (5). It can be used both as elective RT (partial breast irradiation) in selected patients (6) and as an anticipated boost (7). In this case, it shortens the total radiation treatment time by 1–1.5 weeks and improves the precision of dose delivery to the tumor bed (3, 7).

Our Center acquired a mobile electron linac MOBETRON by IntraOp dedicated to IOERT, and our clinical activity started at the end of June 2012.

The risk assessment performed before the start of clinical activity was integrated with the prospective method FMECA (Failure Mode Effects and Criticalities Analysis). Two and four years later, an analysis of all the relevant criticalities was performed in order to improve quality.

One possible approach to prevent failure mode (FM) in IOERT session consists in fact in the identification and prevention of possible hazards *a priori* (8, 9).

The aim of this study is to present the results of the method elaborated by our Working Group and the application of FMECA prospective approach to IOERT procedure.

## Materials and Methods

A multidisciplinary Working Group was created before the beginning of clinical practice with IOERT in 2012, according to the Guidelines for Quality Assurance in Intraoperative Radiation Therapy by the Superior Institute of Health (10), including several different professional profiles: a Surgeon, a Radiation Oncologist, a Physicist, an Anesthesiologist, the Chief of the RT technicians, and the Nurse Responsible for the Operating Block. The Group was coordinated by a facilitator, a Medical Doctor from the Medical Direction of the Hospital, qualified in clinical risk management, skilled in risk analysis, but not expert in radiation therapy.

At first, the Procedure text was elaborated, according to the Joint Commission International (JCI) method (11), followed in our Hospital, with the contribution of all the members of the Working Group, who periodically met with the facilitator.

The description of the Flow Chart of IOERT (Table 1), included in the Procedure text, made up the platform of the FMECA investigation.

**Table 1:** FLOW CHART: processes identified in intraoperative electron beam radiotherapy (IOERT) treatment in Trieste.

Phases	Professional figures	Procedure	Initial risk ranking	Revised risk ranking 2013–2014	Final revised risk ranking 2015–2016
			Risk priority number (RPN)	RPN	RPN
1	Physicist	MOBETRON transfer, start-up and warm-up	45	5	5
			30	10	10
			36	24	24
2	Physicist	MOBETRON daily Mechanical check	30	10	10
3	Physicist	MOBETRON daily dosimetric quality control	60	30	5
4	Nurse of the operating room	Set-up of dedicated IOERT operating table and immobilization device	24	4	4
5	Nurse of the operating room	Preparation of IOERT instruments	12	2	2
6	Anesthesiologist	Quality control of the anesthesiology instruments	12	2	2
			16	4	4
7	Anesthesiologist	Patient monitoring and camera functionality start-up	12	2	2
			16	4	4
8	Nurse of the recovery room	Patient recovery	12	3	3
			8	2	2
			12	3	3
9	Nurse of the operating room	Patient transfer on IOERT operating table	60	30	10
10	Surgeon	Surgical procedure and margin check	45	10	10
11	Surgeon–Nurse of the operating room	Delivery of the specimen of quadrantectomy to the Pathmology Department and margin assessment	60	10	5
			80	10	5
12	Surgeon	Margin assessment communication	45	10	10
13	Surgeon	Preparation of the breast flap	60	10	10
14	Radiation Oncologist–Surgeon	Definition of clinical target volume	64	16	16
15	Radiation Oncologist–Surgeon	Target thickness evaluation	48	24	4
16	Radiation Oncologist	Applicator and shielding disk selection	36	8	8
17	Radiation Oncologist	Dose prescription	45	20	20
		Beam energy selection	45	20	20
18	Physicist	Gafchromic film for *in vivo* dosimetry preparation	60	32	4
19	Radiation Oncologist–Surgeon	Applicator placement	64	36	24
20	Radiation Oncologist–Surgeon	Shielding disk and applicator alignment	80	60	30
21	Radiation oncologist–Surgeon	Applicator connection with docking mirror and support	27	9	9
22	Nurse of the Operating room	Operating table and surgical theater protection with sterilized cover	45	15	15
23	Anesthesiologist	Movement of operating table and anesthesiologist instruments toward the MOBETRON	30	5	5
24	Radiotherapy (RT) technician	Alignment between applicator and MOBETRON gantry	45	30	30
25	RT technician	SOFT-DOCKING	45	10	5
			45	45	30
26	Physicist	Operating room exit	27	12	12
27	Physicist	Mobile shielding set-up	12	6	6
28	Physicist	Monitor unit calculation	45	10	10
29	RT technician	Data entry	45	20	20
30	RT technician	Physical delivery of radiation dose (start button pressed)	48	24	24
31	Anesthesiologist	Anesthesiologist monitoring during irradiation	36	24	24
32	Physicist	Confirmation of dose delivery	60	20	20
33	Physicist	Shielding removal	12	6	6
34	RT technician	MOBETRON removal from the operating table	45	30	30
35	Radiation Oncologist–Surgeon	Removal of all IOERT devices	8	2	2
36	Surgeon	Check of treatment area	20	5	5
37	Radiation Oncologist–Surgeon–Physicist–RT technician	Treatment recording, reporting (in patient report) and signing	36	8	8
38	RT technician	MOBETRON transfer in the pretreatment area and machine switchoff	36	24	24
39	Medical Physics and Clinical Engineering Departments	Machine maintenance	24	12	8

Each member of the Working Group was asked to identify *a priori* the criticalities he/she might meet in the process steps concerning his/her specific activity. In this way, a list of all potential FMs occurring in each process step was drafted.

FMECA leads to quantitative data: for every step of IOERT procedure a score is assigned. The score ranges from 1 to 5 and includes three pre-established parameters:

– severity (S),
– occurrence (O),
– detectability (D).

In clinical practice, FMECA consists in the identification of different steps of the examined procedure (12). For each step, possible critical situations are identified and a risk priority number (RPN) is assigned. RPN value is obtained by multiplying S, O, and D parameters (RPN = S × O × D) (score between 1 and 5 assigned following rules) (Table 2).

**Table 2:** Risk Analysis.

Severity (S)	Occurrence (O)	Detectability (D)
1.No damage	1.Extremely unlikely	1.Almost always detected
2.Minimal damage	2.Low probability	2.Great probability to be detected
3.Moderate damage in the short term	3.Moderate probability, it occasionally occurs	3.Moderate probability to be detected
4.Main damage in the long term	4.Great probability, it repeatedly occurs	4.Low probability to be detected
5.Permanent damage	5.Very high probability, almost inevitable	5.Very low probability to be detected, remote

The higher the value obtained, the more likely the risk that an accident occurs (“failure”) during the procedure and the higher the probability of relevant consequences are.

Based on calculated RPN, four different “risk classes” are identified:

– low risk (RPN ≤30),
– moderate risk (RPN 31–40),
– intermediate risk (RPN 41–50),
– high risk (RPN >50).

Thus, the identification of the critical steps of a procedure leads to modifications, even of substantial nature, of behavior and actions in order to reduce errors occurrence as much as possible. Decreasing this hazard also leads to the reduction of possible damage both for health personnel and patients.

The risk analysis was completed by asking the members of the team to evaluate the RPN of each FM.

Every 2 years since the beginning of IOERT clinical activity, the risk analysis was repeated by the Working Group in order to assess the improvement achieved.

## Results

Our activity with IOERT as an anticipated boost, followed by conventional or hypofractionated external beam RT, in the treatment of early breast cancer started in June 2012.

Ninety-two cases have been treated up to April 2016 (46 by the end of 2014 and an additional 40 by the end of 2016).

The first FMECA analysis was performed before the start of clinical activity; the risk analysis was then repeated at the end of 2014 and again at the end of 2016.

In the Flow Chart, the IOERT process was subdivided into 39 steps, in which the different professional figures are involved, from the start-up of the Mobetron to the end of the operation, the switch off of the machine and the draft of the IOERT report (Table 1).

An Excel worksheet was created, inserting in rows: process step, professional figures involved, FM, potential effects of failure, potential causes of failure, initial risk ranking with the RPN, and corrective actions. In the re-analysis of the process—2 and 4 years later—the final RPN was elaborated and the risk reduction (RR) (preliminary RPN—final RPN) was also calculated in order to assess the weight of the corrective measures.

In this worksheet, 51 FMs were identified, which represented the issues prospectively investigated according to the FMECA method.

Considering a set threshold of 30, the evaluated RPNs show that 33 out of 51 FMs are critical; 6 are included in the moderate risk class (RPN: 31–40); 16 in the intermediate risk class (RPN: 41–50), and 11 in the high risk class (RPN: >50).

Failure modes included in the high risk class (Table 3) enabled us to pinpoint the main criticism of the whole IOERT procedure and so we were able to make procedural changes in order to reduce hazards. The data highlight the fact that several critical steps concern the surgical procedures and IOERT set-up.

**Table 3:** FMECA worksheet: high risk class steps.

Phases	Professional figures	Procedure	Failure mode	Failure effects	Failure causes	Initial risk ranking				Corrective actions 2013–2014	Revised risk ranking				Corrective actions 2015–2016	Final revised risk ranking			
						S	O	D	Risk priority number (RPN)		S	O	D	RPN		S	O	D	RPN
3	Physicist	Pre treatment quality control and authorization	Wrong measure, insertion or reading of a delivery parameter	Wrong delivered dose	Violation of protocol limits for quality assurance	5	3	4	60	Adherence to instructions in quality assurance protocol	5	2	3	30	Adherence to instructions in quality assurance protocol—wider experience	5	1	1	5

9	Nurse	Patient positioning on the operating table	Patient displacement	Patient displacement, slipping and fall off	Non-adherence to guidelines/protocol	5	4	3	60	Adherence to Hospital Policies and Procedures	5	2	3	30	Adherence to Hospital Policies and Procedures—wider experience	5	1	2	10

11	Surgeon–Nurse	Delivery of surgical specimen to the Pathology and/or Radiology Department	Transcription error	Wrong patient identification	Communication defect	5	4	3	60	Procedure adherence	5	2	1	10	Procedure adherence-wider experience	5	1	1	5
				Wrong therapeutic decision	Communication defect	5	4	4	80	Surgeon confirmation required	5	2	1	10	Procedure adherence-wider experience	5	1	1	5

13	Surgeon	Breast flap preparation	Tissue devitalization	Post operatory morbidity-reintervention	Fat necrosis	5	4	3	60	Careful visual control of tissue vitality	5	2	1	10	Careful visual control of tissue vitality	5	2	1	10

14	Radiation Oncologist–Surgeon	Treatment area definition	Wrong treatment area definition	Wider or smaller treated area	Wrong visual evaluation of the tumor bed to be irradiated	4	4	4	64	Preventive evaluation of tumor dimensions (Mammography, Ultrasound, MRI); intraoperative evaluation	4	2	2	16	Preventive evaluation of tumor dimensions (Mammography, Ultrasound, MRI); intraoperative evaluation	4	2	2	16

18	Physicist	Preparation of gafchromic film and placement on the shielding disk	(1)Inadequate placement (2)Wrong calibration, use, conservation of the gafchromic film	Wrong measure of the delivered dose	Wrong observance of the “*in vivo* dosimetry” procedure	4	3	5	60	Observance of the “*in vivo* dosimetry” procedure	4	2	4	32	Observance of the “*in vivo* dosimetry” procedure, labeling of the gafchromic film and double check	4	1	1	4

19	Radiation Oncologist–Surgeon	Applicator placement	Absent or incomplete adherence of the applicator to the tumor bed	Non-homogeneous irradiation	Air gap presence, blood accumulation, very curved tumor bed	4	4	4	64	Accurate visual control, correct placement of the patient on the operating table	4	3	3	36	Accurate visual control, correct placement of the patient on the operating table, double check	4	3	2	24

20	Radiation Oncologist–Surgeon	Alignment of the shielding disk	Misalignment of the shielding disk	Unintended normal tissues irradiation below the tumor bed	Low accuracy in the alignment	5	4	4	80	Selection of a disk much larger than the applicator size	5	3	4	60	New shielding set-up and check with the ultrasound probe	5	3	2	30

32	Physicist	Verification of the correctness of the parameters related to the delivered treatment	(1) Transcription error (2) Late data registration (information loss)	Wrong evaluation of the delivered treatment	Oversight or non-observance of the procedures	5	3	4	60	Check list and double check between Physicist and Radiation Oncologist	5	2	2	20	Check list and double check between Physicist and Radiation Oncologist	5	2	2	20

## Discussion

The analysis of adverse events in RT is a recent topic; either retrospective or prospective approaches can be employed. In the former group, the root cause analysis is the most widely used, aimed at identifying the root causes of near misses or adverse events; in the latter group, several methodologies are available, such as process mapping, value stream mapping, fault tree analysis and failure mode effects and criticality analysis (FMECA) (12). A proactive approach allows to study the whole process or part of it, independently from the occurrence of an adverse event, to implement the corrective actions and to evaluate the benefits obtained; it is, therefore, more suitable to a complex process, such as IOERT.

Analyzing the high risk class steps of our procedure in chronological order (Table 3), the first phase with a high risk score (RPN = 60) concerns the Physicist who is in charge of the program of quality control (*FMECA step No. 3*).

During the pre-clinical quality control, the FM was due to the incorrect measurement and/or reading of the pre-established parameters, which might cause the delivery of a wrong dose to the patient. The precise observation of the Quality Control Protocol at first (RR: 30; 50%) and then a wider experience of the operator (RR: 25; 83.3%) could avoid this serious occurrence.

The second criticality involves the nurses of the Operating Theater (*step No. 9*): it concerns the incorrect positioning of the patient on the operating table with the danger of slipping or falling off (RPN = 60). The adherence to the well-known Policies and Procedures of our Hospital (RR: 30; 50%) and the training of the nursing staff acquired over time (RR: 20; 66.7%) could prevent these accidents.

In the FMECA analysis, a high score was also assigned to the phase *No. 11*, which involves both the operating room Nurses and the Surgeon; it regards the delivery of the specimen of quadrantectomy to the Pathology Department and the margins assessment with a macroscopic evaluation performed by the Pathologist.

Assuming that the patient identification (*step No. 11a*; RPN = 60) has been properly done during the “time out” phase (before the surgical procedure begins) (final RPN: 5), the main debated issues are as follows: the way to communicate sensitive data (by phone), the amount of time to obtain the results, and the right evaluation of the Pathologist report. Time delay for surgical specimen examination may vary from 15 to 30 min due to the time for mammographic/ultrasound evaluation of the specimen, sample processing, and caseload of the Pathologists.

A wrong identification of the margin (*step No. 11b*; RPN = 80) could cause an incorrect choice of the surgical procedure; the risk of local failure in fact is reported to be higher in case of positive or close margins (13) and, in this case, the Surgeon can widen them during the same surgical procedure.

We identified and selected a group of Pathologists specialized and dedicated to breast tumors, with the aim to reduce this risk ranking. As regards sensitive data communication, a specific procedure was established: data need to be communicated by phone from a physician to another physician and the colleague who receives the communication has to print the online report in order to have an official written document (final RPN: 5).

*Step No. 13* involves the Surgeon (RPN = 60) and the correct preparation of the breast flap in order to prevent that a devitalized tissue could cause necrosis and postoperative morbidity. An accurate visual control during the surgical procedure could decrease the risk (RR: 50; 83.3%).

*Step No. 14* regards the definition of the clinical target volume (CTV) (RPN = 64). This represents a critical point involving the Radiation Oncologist and the Surgeon: underdosing of the target and/or unintended normal tissues irradiation can occur (14). The exact evaluation of the dimensions of the tumor on preoperative diagnostic imaging and an accurate intraoperative definition of the CTV determined a significant RPN reduction from 64 to 16 (RR: 48; 75%).

Another relevant criticality (*step No. 18)* involves the Physicist and the preparation of the gafchromic film for *in vivo* dosimetry (RPN = 60). A wrong calibration of the gafchromic film or an inadequate placement of the film on top of the internal shield can cause a wrong evaluation of the dose delivered (14). These risks were prevented first of all by following the “*in vivo* dosimetry” Procedure, elaborated by the Physicist, with an RPN reduction from 60 to 32 (RR: 28; 46.7%), and then by labeling the gafchromic film and employing a double check in the procedure with a further reduction of RPN from 32 to 4 (RR: 28; 87.5%).

The next critical score (*step No. 19)* is related to the inaccurate placement of the applicator in the tumor bed (RPN = 64).

Absent or incomplete adherence of the applicator to the tumor bed, determined by air gap presence, blood accumulation, or very curved tumor bed, can cause a non-homogeneous irradiation (14). Corrective actions, such as an accurate visual control and the correct placement of the patient on the operating table, reduced the risk ranking from 64 to 36 (RR: 28; 43.8%) after 2 years and from 36 to 24 in the next 2 years (RR: 12; 33.3%) employing a double check.

**Figure 1:**
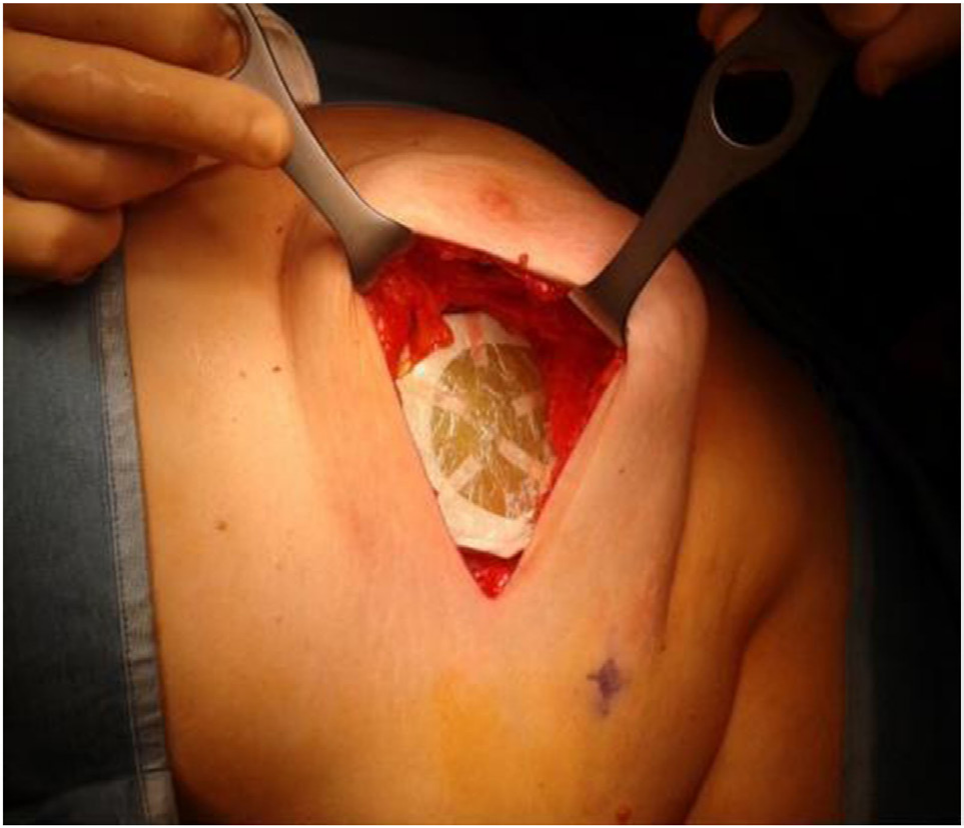
Disk placement behind a patient’s breast parenchyma before IOERT.

The highest step score (*No. 20)* (RPN = 80) was attributed to the misalignment of the shielding disk, used to protect the normal tissues underneath the target volume, such as the lung and the heart (for the left breast). The disk is positioned by the Surgeon between the residual breast and the pectoralis fascia, its size depending on the collimator diameter chosen for the treatment (Figure 1).

**Figure 2:**
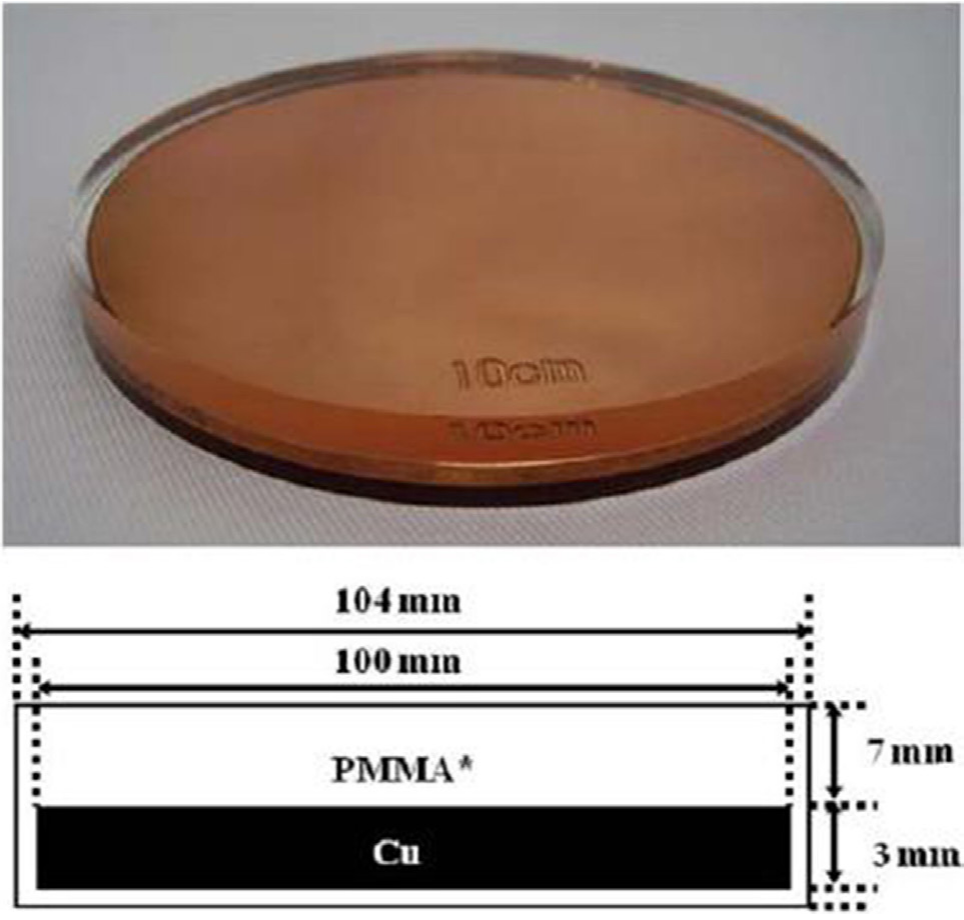
Sketch of the shielding disk, showing the stack of PMMA and copper layers.

In most of the cases, we employed an 8-cm-diameter shielding disk provided by the IntraOp, made up of three stacked layers: a 5 mm polymethyl methacrylate (PMMA), a 3 mm copper, and a 2 mm PMMA layer (Figure 2).

The low accuracy in the alignment of the disk would cause the delivery of an excess of dose to the underlying normal tissues.

The selection of a plate much larger than the applicator size was the first corrective action.

**Figure 3:**
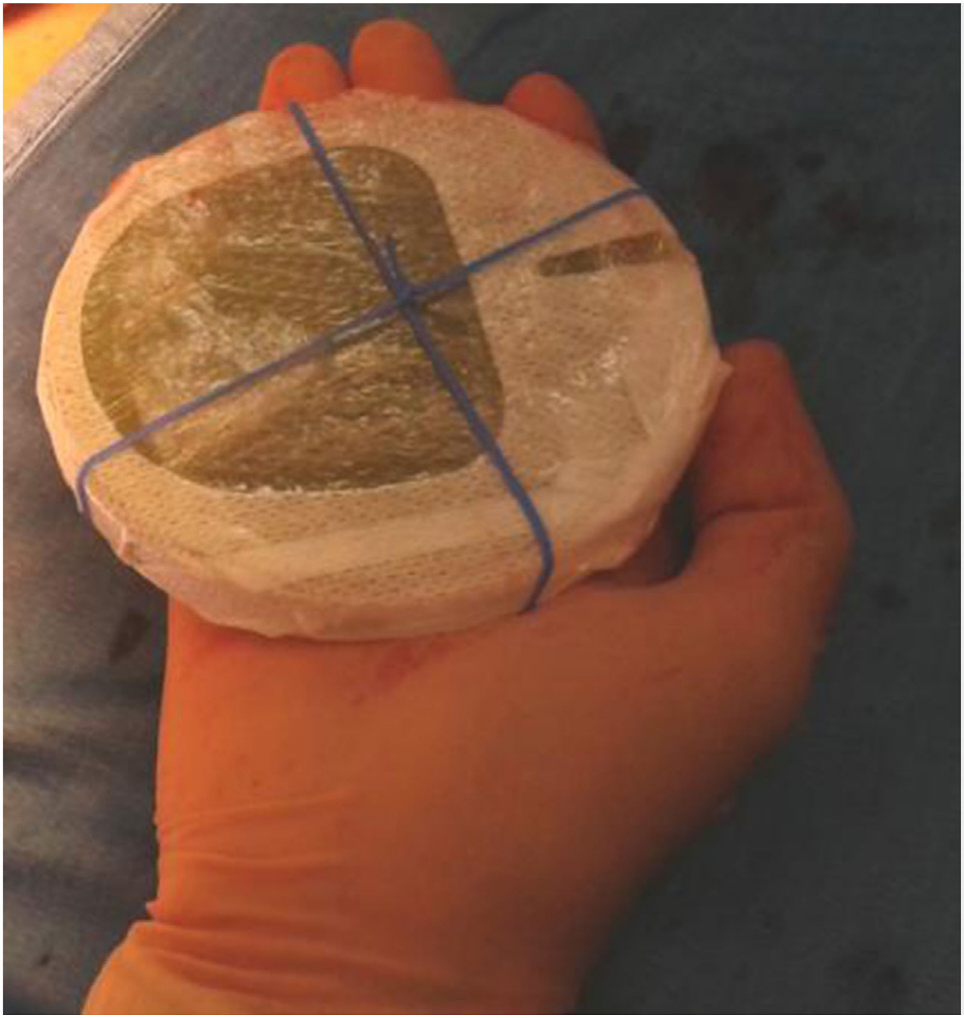
New shielding set-up with the surgical elastic band.

Furthermore, to avoid shielding disk misalignment, the Surgeon and the Radiation Oncologist decided to design a new shielding set-up, implementing an elastic band fixed by stitches to the pectoralis fascia, thus preventing the disk slipping (14) (Figure 3).

The optimized set-up described above was introduced into the clinical practice, and a RR from 80 to 60 (RR: 20; 25%) was observed in the first re-analysis of the procedure in 2014.

In 2015, intraoperative ultrasound (IOUS) measures were employed either to better define the target thickness or to check the position of the shielding disk. IOUS proved to be accurate in evaluating both target thickness and shielding alignment, halving the RPN (RR from 48 to 24 in the former case and from 60 to 30 in the latter case).

The last high risk step in the FMECA worksheet (RPN = 60) concerns the Physicist and the verification *a posteriori* of the parameters of the delivered dose *(step No. 32)*. The constant use of the check list and a double check by the Physicist and the Radiation Oncologist contributed to decrease the RPN from 60 to 20 (RR: 40; 66.7%).

Only two studies published in the literature examined the importance of FMECA applied to IOERT procedure: in the Italian experience a dedicated mobile Linac was used (8), while in the Spanish analysis the irradiation was performed with a fixed conventional Linac (9). In both studies, FMECA proved to be an excellent tool to evaluate patient safety and allowed to reduce risks and improve quality in IOERT procedure. Ciocca et al. (8) found that the highest risk was associated with the alignment of the shielding disc, as we observed in this study (Table 3). López-Tarjuelo et al. (9) found that the highest ranked RPNs were related not only to incorrect protection assembly but also to incorrect transmission and programming of treatment parameters. In our analysis, incorrect monitor unit calculation and data entry (step No. 28 and No. 29) (Table 1) had an initial intermediate risk score, which decreased to a low score after the introduction of the corrective actions, while only the verification *a posteriori* of the delivered dose (step No. 32) was initially classified in the high risk class, as reported above.

## Conclusion

Intraoperative radiation treatment as an anticipated boost proved to be very effective in the treatment of early breast cancer.

This approach presents several advantages:

– No topographic miss,
– More favorable radiobiology of a single dose (α/β),
– Shorter radiation time (<1–2 weeks),
– Good dose distribution,
– Complete skin sparing,
– Minimal toxicity in the long-term follow-up.

On the other side, it reveals some drawbacks:

– Uncertainty of the final pathologic report and
– Lack of definition of the resection margins.

To date, every published interim analysis showed lower local recurrence rates than standard treatment schedules (7). In our experience, no local or regional recurrences were detected, with a median follow-up of 28 months, and the incidence and severity of the side effects were acceptable; no grade 3 or greater side effects were observed.

The FMECA has provided a prospective systematic method to discover potential failures in IOERT procedure: evaluating not only the frequency of FM but also their severity and detectability, it has given a more complete assessment of the risk. It contributes, therefore, to optimize patient safety right from the start of our clinical activity and to improve risk management culture among all the professionals involved in the Working Group.

The IOERT procedure was standardized and the application of FMECA allowed us to define the safest pathway for the patient.

Its use, still rather limited, should be strongly encouraged in RT Centers treating patients with IOERT.

## Author Contributions

The right sequence of the names is as follows: CV, MS, MU, LT, AP, and MB (corresponding Author). CV, MS, and MB elaborated the test; MU and LT analyzed the patient data; and AP gave management support.

## Conflict of Interest Statement

The authors declare that the research was conducted in the absence of any commercial or financial relationships that could be construed as a potential conflict of interest.
